# Association between Physical Fitness and Low Back Pain: The Pepe Cross-Sectional Study

**DOI:** 10.3390/children9091350

**Published:** 2022-09-04

**Authors:** Aina M. Galmés-Panadés, Josep Vidal-Conti

**Affiliations:** Physical Activity and Sport Sciences Research Group (GICAFE), Institute for Educational Research and Innovation (IRIE), University of the Balearic Islands, 07122 Palma, Spain

**Keywords:** physical fitness, low back pain, physical activity, schoolchildren

## Abstract

Background: Recent studies have shown that the lifetime prevalence of low back pain (LBP) in schoolchildren aged 10–12 years is 73.6%, and that it appears to have an impact on people’s quality of life. A wide range of risk factors associated with LBP have been studied. However, inconsistent results have been reported. In recent decades, the physical fitness level of children and adolescents has worsened, and the current data on the relationship between muscular fitness and musculoskeletal pain are ambiguous. The purpose of the present study was to analyze the relationship between physical fitness and the occurrence and intensity of LBP. Methods: This cross-sectional study assessed 849 students, aged 10–12 years, from 10 primary schools (fifth and sixth grades) from Majorca (Spain). It was based on two different structured and self-administered questionnaires and a fitness test battery validated for child populations. Results: The results showed that higher levels of VO2Max correspond to less LBP intensity. Additionally, LBP was less prevalent among participants who self-reported more physical activity, and higher VO2Max and higher levels of flexibility were associated with the absence of LBP in bed. Conclusion: These results are of particular importance, as cardiorespiratory fitness is the parameter most closely related to health, and it seems to also be related to LBP-prevention.

## 1. Introduction

Recent studies have shown that the lifetime prevalence of low back pain (LBP) in children aged 6–12 years is 47.5% [1]; the lifetime prevalence in children aged 10–12 is 73.6% [2]; and the one-year prevalence among children aged 10–19 is 41.5% [3]. It seems that LBP affects people’s quality of life, and it may even impair their ability to go about their daily activities [4].

Knowing which risk factors are associated with back pain is important in order to design prevention strategies among young people to reduce the burden of LBP. A wide range of risk factors has been studied, including sociodemographic variables (such as age or educational level), biological variables (such as weight, body mass index or sex), psychosocial variables (such as depression or societal relationships), and lifestyle-related variables (such as physical activities, screen time, ergonomics), as potential risk factors of LBP in children [5,6,7]. Additionally, the most common spinal disorder in children and adolescents is scoliosis [8]. In particular, adolescent idiopathic scoliosis (AIS) has been identified as a potential risk factor for the development of LBP in adolescents [9]. However, inconsistent results have been reported regarding these risk factors and their relationships with LBP in children and adolescents.

In the present study, we focus on two variables related to lifestyle, which are physical activity (PA) and physical fitness. The relationship between PA and LBP seems to be curvilinear in adolescents, given that both low and high PA levels are associated with an elevated risk of LBP [10]. Additionally, certain types of PA have also been linked to the risk of LBP in adolescents, such as gymnastics, wrestling, diving, rowing, and American football [11].

There are enough data in the literature to support the benefits of muscular exercise on bone, cardiometabolic, and mental health. However, data on the impact of muscle fitness on musculoskeletal pain are limited to the link between trunk extensor and flexor endurance and LBP [12]. According to several studies, the data on the relationship between muscular fitness and musculoskeletal pain are ambiguous [13]. 

Health-related physical fitness comprises cardiorespiratory fitness, muscle strength/resistance, flexibility, and body composition [14]. To date, no studies have examined the relationship between objectively measured cardiorespiratory fitness and LBP in children, although a study found aerobic capacity to be unrelated to undifferentiated neck/back pain [15].

In recent decades, the physical fitness levels of child and adolescent populations have worsened, especially the cardiorespiratory fitness and muscular power indicators [16]. The need to improve the adherence of child and adolescent populations to PA recommendations is evident, in order to improve cardiorespiratory fitness, and to prevent and treat multiple health complications [17]. The main hypothesis of the present study was that high levels of PA and physical fitness will be associated with less LBP, both in terms of the pain frequency and intensity. The aim of the present study was to analyze the association between the levels of PA and physical fitness with LBP, in terms of the pain frequency and intensity.

## 2. Materials and Methods

### 2.1. Participants

**Figure 1:**
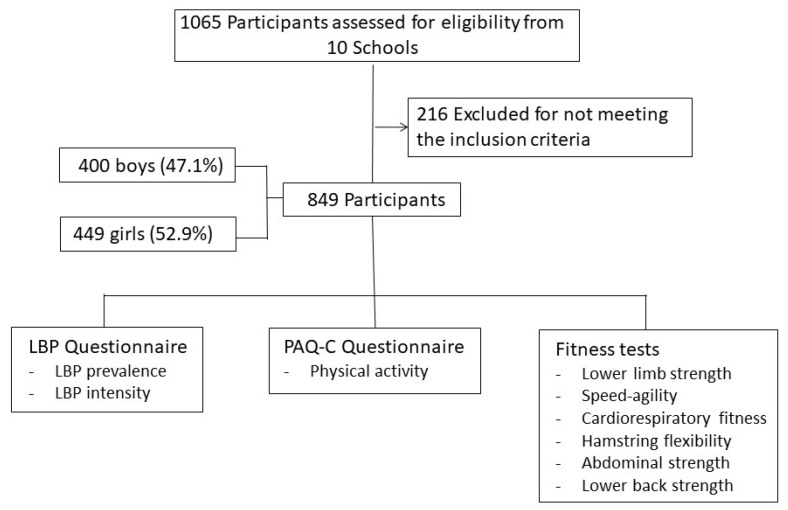
Flow chart.

This cross-sectional study assessed students aged 10–12 years from primary schools (5th and 6th grades) in Majorca (Spain), nested in the PEPE randomized controlled trial (Spain) [18]. Data collection was carried out between February and March 2021. This study was conducted using a final sample of 849 participants from 10 primary schools (Figure 1). According to the formula S = ((z^2^ xp (1 − p))/e^2^)/ = (1 + ((z^2^xp (1 − p)/e^2^N)), where S = sample size, N = population size, e = margin of error, z = z-value, and a reliability level of 95%, the sampling error was 3.4%. Of these 849 participants, 400 were boys (47.1%) and 449 were girls (52.9%). The mean age was 11.3 (33.5%, 47.9%, and 18.6% were 10, 11, and 12 years old, respectively). The sample was chosen by convenience selection from various clusters (schools). An invitation to participate in the study and information about its goals and characteristics were sent to all schools in a letter.

### 2.2. Selection Criteria

As the inclusion criteria, the students must be aged between 10 and 12 years old and be in the 5th or 6th grade of primary school. The following were considered as exclusion criteria: the students whose parents or tutors did not sign and return the informed consent form, and those who did not participate due to illness or disability.

### 2.3. Instruments

This study was based on two different structured and self-administered questionnaires and a fitness test battery for children. In addition, self-reported height and weight were included in the questionnaires to determine the body mass index (BMI).

A validated questionnaire [19] was used to collect data on LBP, including the lifetime prevalence, prevalence in the last seven days (yes/no), point prevalence (yes/no), LBP intensity over the last three months (visual analogue scale—VAS—which ranged from 0 to 10), LBP-impeding usual activities (never/only when in pain/always), LBP in bed or upon rising (yes/no), sex, and age. The prevalence of LBP (LBP ever) was assessed through the question “Have you ever had low back pain?”. The answer options were (1) never; (2) only once; (3) several times; (4) frequently; (5) almost constantly. Due to the self-reporting nature of the questionnaire and the age of the participants, we decided that reporting LBP “only once” was not representative of suffering from LBP, as it is a singular occurrence, so the variable was categorized as dichotomous, with 0 being never having experienced LBP or having LBP only once, and 1 being having experienced LBP several times, frequently, or almost constantly.

The Physical Activity Questionnaire for Children (PAQ-C) [20] was used to assess the physical activity levels. The Spanish version [21] was used in the present study. This self-report questionnaire comprises nine items. It is a seven-day PA recall questionnaire to assess the participants’ PA during free time, physical education classes, recess, at lunch, right after school, in the evening, and on weekends. The score of each item was recorded along a five-point Likert scale from low PA (1) to high PA (5). The final PAQ-C score is the mean score of all the items. 

The ALPHA (Assessing Levels of Physical fitness and Health in Adolescents) health-related fitness test battery for children was used to measure the cardiorespiratory fitness, speed and agility, and lower-limb muscular strength [22]. The lower-limb muscular strength was assessed through the standing long jump test. The objective is to jump as far as you can from a starting point behind a line. The best value of two attempts was recorded in centimeters. Speed and agility were assessed through the 4 × 10 m shuttle run test. It consists of running back and forth between two lines, 10 m apart, as quickly as possible. The minimum time (in seconds) taken to complete the test from two attempts was used in the analyses. For analytic purposes, the values were multiplied by −1, so a higher score denotes more speed and agility. The cardiorespiratory fitness was assessed by the Course Navette test (20 m shuttle run test). It consists of running back and forth between two lines 20 m apart for as long as possible. Audio signals controlled the tempo. When the children were unable to cross the lines twice before the sound signal, the test was over. The number of 20 m laps was recorded and used in the analyses. Based on the Leger’s equation, applied to children between the ages of 6 and 17.9, the maximal oxygen volume (VO2max) was calculated: [VO2 max = 31.025 + (3.238 × Speed) − (3.248 × Age) + (0.1536 × Speed) × Age].

The children’s hamstring flexibility was evaluated using the sit-and-reach test [23]. Both legs were extended and the participant’s feet were flat against the box while they were sitting on the floor. The participant held the final position for at least two seconds while slowly reaching forwards with both hands, one on the other, as far as they could without jerking. The final location of the fingertips on or towards a ruler was recorded as the test score in centimeters. The score was negative if the participant could not contact the box’s front with their fingertips, where the “0” score is. The best maintained value was used after the test was run twice.

Abdominal strength was assessed by performing static curl-ups following a modified version of the FitnessGram curl-up test. Participants were asked to keep their fingers at the end of the 12 cm wide stripe as long as they could, while maintaining the “up” position (body at 45°, arms outstretched). If their fingers strayed from the stripe’s edge, they had three seconds to return to the proper position before the test was declared failed. Analysis was done using the total amount of time they spent holding the proper position [24]. 

Lower-back strength was assessed by the Biering–Sørensen test [25]. The starting position was a prone position on a table with the lower limbs and the pelvis supported and the upper body extending beyond the table’s edge. The test consisted of holding the upper body in a horizontal position for as long as possible. The test was conducted only once, without a warm-up or practice, and the results were recorded in seconds.

In addition, a total test score was calculated to represent the children’s overall performance in one score. The fitness score was created by adding up the six fitness tests (z-score)—hamstring flexibility, speed–agility, abdominal strength, lower-back strength, lower-limb muscular strength, and VO2Max—and dividing the result by six [26]. The higher the score, the better the children’s physical fitness, and the lower the score, the worse their physical fitness.

### 2.4. Procedure

The questionnaires were administered at home or at school. All participants (students, teachers, and parents) were made aware of the goal and methods of the study. The teachers administered the questionnaires in the school’s classroom using laptops, or provided the families with a guide to complete them. The questionnaires were accessible using Google Forms. Moreover, the parents or tutors of the students were asked for their approval before their children could take part in the study. 

The fitness test data were collected by expertly trained teams of 3–4 people, who went to each school to administer the fitness test battery.

The study was approved by the Research Ethics Committee of the University of the Balearic Islands (reference number: 130CER19).

### 2.5. Statistical Analysis

Participants were classified into two categories, depending on the presence or absence of LBP. The first category includes the participants who suffer from LBP (Yes), and the second category includes those participants without LBP (No). The standard deviations (SDs) and means were used to summarize the descriptive characteristics, as well as the numbers and percentages (%). A one-way analysis of variance (ANOVA) and Chi-square test (χ^2^) were used to assess the differences between the groups which experience LBP for continuous and categorical variables, respectively.

Multivariate linear regression analyses were conducted to estimate the β-coefficients and 95% confidence intervals (CIs) for the associations between the intensity of LBP (outcome variable) and height, body mass index (BMI), PAQ-A score, and physical fitness—specifically flexibility, speed, abdominal strength, lower-back strength, horizontal jump, and VO2Max. Our main model was adjusted for age, sex, and school. Logistic regression models were used to assess the association between the categories of physical fitness and pain frequency: ever, in the last 7 days, today, and in bed. The logistic regression models were adjusted for the age, sex, and school. The exposure variables (flexibility, speed, abdominal strength, lower-back strength, horizontal jump, and VO2Max) were categorized into two categories, using the 50th percentile as the cut-off point. In the present analyses, category 1, below the 50th percentile, was taken as the reference category, and the results corresponding to category 2, above the 50th percentile, are shown. In the case of the outcome variables, the categorization was carried out based on the presence or absence of pain; the values shown correspond to no pain, and the reference categories contained participants who suffered from pain. A bar chart with error bars was created to assess the association between each item of physical fitness (z-score) and the fitness score (z-score) with the prevalence or absence of LBP. The percentage of missing values across the variables varied between 0 and 21%. The participants with item-level missing data were excluded and only the available items were analyzed.

The statistical analyses were performed using the Stata v17.0 program. *p*-values < 0.05 were considered as statistically significant.

## 3. Results

Table 1 describes the characteristics of the population, classifying it into two groups according to the presence or absence of LBP. The population group with LBP was slightly older, and the women in this group had a higher height and body weight on average. On the contrary, the group without LBP was more physically active.

Table 2 shows the association between physical fitness and LBP intensity and prevalence. Participants with higher VO2Max experienced lower-intensity LBP (*p* = 0.022). In the group of participants who self-reported more PA, determined via their PAQ-A-score, a lower prevalence of LBP was found, but, in this case, the association was borderline significant (*p* = 0.078). No differences were found between the boys and girls.

Table 3 shows the odds ratios for the prevalence of LBP: ever, in the last 7 days, today, and in bed. This is organized by the categories of physical fitness and PA. Participants who, according to the validated PAQ-A questionnaire, were more physically active had no LBP ever (*p* = 0.004). Those participants with higher VO2Max and hamstring flexibility had no LBP in bed (*p* = 0.049 and *p* = 0.020, respectively). No further significant associations were observed. Additionally, no differences were found between the boys and girls.

**Figure 2:**
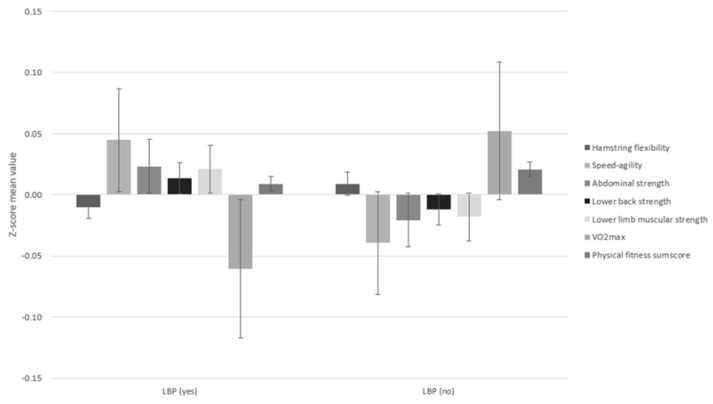
Individual physical test items by LBP lifetime prevalence.

The total fitness score for each child was calculated as the average of z-scores for the individual physical test items. Figure 2 shows a bar chart with error bars of the total fitness score, and each physical test separately against the presence or absence of LBP ever.

## 4. Discussion

The results from this cross-sectional study show that having a higher VO2Max was associated with lower LBP intensity in a sample of schoolchildren. In addition, performing more PA was associated with the absence of LBP, and higher VO2Max and higher levels of flexibility were associated with the absence of LBP in bed.

The present results show that higher levels of aerobic capacity, measured using VO2Max, were associated with the absence of LBP and lower LBP intensity. No significant associations were found with the strength variables (abdominal strength, lower-back strength, and lower-limb muscular strength), nor with the speed–agility test. Due to the lack of an association between the abdominal, lower-back and lower-limb muscle strength and LBP intensity and frequency, controversy exists in the current literature. Some studies found no association between the back muscle strength and LBP in adolescents [27], nor between the performance when doing push-ups and LBP in the child and adolescent populations [28]. On the other hand, some cross-sectional studies found that the reduced endurance of the trunk extensor muscles [29,30], as well as the trunk flexor muscles [31], was associated with LBP. Moreover, strengthening exercises were found to be effective for reducing pain and improving back function in the adult population [32]. In terms of lower-limb endurance and power, some studies found an association between higher endurance [29,30] and power [33] with less LBP, but, in the present study, no associations were found. Moreover, research suggests that core-conditioning intervention programs improve lower-back injuries by improving the core muscle strength and, therefore, overall trunk stability [34,35,36,37], but no association was found in the present study between the abdominal strength and LBP. Some authors point out that the mechanisms that explain how muscle strength acts to compensate for external loading, leading to back pain symptoms, remain unknown [38], probably due to the lack of prospective studies, which does not allow causality to be established [12].

While it is true that there are many studies analyzing fitness associated with health [24,39,40,41], few studies have investigated the association between the fitness level and the LBP [42,43,44], especially in child populations [45,46,47]. In the present study, higher levels of PA were associated with the absence of LBP. These results are in line with previous studies, in which low levels of PA were found to increase the risk of LBP in schoolchildren [48], and higher levels of PA were associated with lower levels of LBP in adolescents [47]. A systematic review and metanalyses found an inverse association between PA and LBP [42]. However, other systematic reviews suggest that the body of evidence on the role of the PA level and low back pain is somewhat inconsistent [49]. As noted in a systematic review, one of the possible explanations for the inconsistency of results across studies may be related to the heterogeneity in the exposure assessment methods across studies [49]. Other studies found no association between PA, measured with an accelerometer, and LBP in children and adolescents, neither at the cross-sectional level nor after two years of follow-up [50]. In the present study, the PA was self-reported with the validated PAQ-A questionnaire, which presents a limitation, but, on the other hand, the fitness was measured with standardized physical tests, which ensures the objective measurement of physical fitness and allow comparisons with other studies. In relation to the respiratory fitness, which was measured with the Course Navette test, the VO2Max was estimated. In future studies, the use of accelerometers will be recommended to objectively measure PA. In the present study, the VO2Max was associated with lower LBP intensity and the absence of LBP in bed, which is in line with the results of the PA practice from the present study. These results are of particular importance, as cardiorespiratory fitness is the parameter most closely related to health [51]. 

As for the physical fitness score, in order to assess physical fitness more globally, a score combining flexibility, muscular endurance, muscular strength, aerobic capacity, balance, and speed was created. No association was found between a better fitness score and lower LBP, which is consistent with another study [52].

As mentioned throughout the discussion, there is a lack of evidence on which fitness factors influence LBP, and how the onset of LBP can be prevented and delayed. As other authors have noted, the lack of consistency in the results may be due to the lack of studies, the risk of bias, and the low quality and objectivity of the evidence [52].

A marked strength of this study was the use of a large cohort of schoolchildren, as well as the use of validated questionnaires and validated physical tests. This decreases any potential bias or measurement error and expands the window for literature comparison. As limitations, the cross-sectional design’s shortcomings include the inability to determine causality. We cannot exclude reverse causality because the exposure and outcome variables were measured simultaneously. Moreover, the measurement of PA was self-reported; for future studies, the use of an accelerometer should be considered.

## 5. Conclusions

In conclusion, the results from this study indicate that, in a child population aged 10–12 years, higher levels of VO2Max are associated with a lower intensity of LBP and absence of LBP in bed. Moreover, higher levels of PA are associated with the absence of LBP, and higher levels of flexibility are associated with the absence of LBP in bed.

## Figures and Tables

**Table 1:** Study population’s baseline characteristics across categories of frequency of lifetime prevalence of low back pain.

	Lifetime Prevalence of Low Back Pain			
	Total n	Yes	No	*p*-Value
Age, years	797	11.3 (0.69)	11.2 (0.67)	0.038
Women, n (%)	443 (52.9)	231 (59.8)	212 (47.0)	<0.001
Men, n (%)	394 (47.1)	155 (40.2)	239 (53.0)	<0.001
Anthropometric measures				
Weight (kg)	770	43.0 (10.4)	41.6 (9.24)	0.051
Height (cm)	749	151 (9.19)	149 (8.70)	0.017
BMI (kg/m^2^)	708	18.9 (3.61)	18.7 (3.56)	0.392
Physical Activity Habits				
PAQ-A-Score	837	2.98 (0.63)	3.08 (0.64)	0.019
Physical Fitness				
Hamstring flexibility ^1^	706	−1.40 (9.09)	−1.22 (9.68)	0.801
Speed-agility ^2^	713	22.9 (102)	16.1 (56.3)	0.262
Abdominal strength ^3^	706	107 (78.7)	103 (76.6)	0.561
Lower back strength ^4^	713	103 (67.5)	101 (71.6)	0.733
Lower limb muscular strength ^5^	673	140 (26.8)	139 (26.1)	0.614
VO2 Max ^6^	832	42.1 (6.23)	42.8 (7.26)	0.104

Data shown is mean (SD), unless otherwise specified. Abbreviations: BMI: body mass index; VO2Max: maximum oxygen volume. Data on low back pain were absent for 6 boys and 6 girls. Test (^1^) Hamstring flexibility was assessed by sit-and-reach. Test (^2^) Speed–agility was assessed with the 4 × 10 m test. Test (^3^) Abdominal strength was assessed by the test of trunk muscular endurance. Test (^4^) Lower back strength was assessed by the Biering–Sørensen test. Test (^5^) Lower limb muscular strength was assessed by horizontal jump test. Test (^6^) VO2Max was estimated by the Course Navette field test, following the ALPHA battery.

**Table 2:** Associations of physical fitness with low back pain.

Exposures	Outcome (Intensity of Low Back Pain)		Outcome (Low Back Pain Prevalence)	
	SC β (95% CI)	*p*-Value	SC β (95% CI)	*p*-Value
Anthropometric measures				
Weight (kg)	−0.00 (−0.02; 0.02)	0.961	0.03 (−0.00; 0.01)	0.404
Height (cm)	0.00 (−0.02; 0.02)	0.977	0.07 (0.00; 0.02)	0.065
BMI (kg/m^2^)	0.00 (−0.05; 0.05)	0.937	−0.00 (−0.02; 0.02)	0.930
Physical Activity Habits				
PAQ-A-score	−0.04 (−0.43; 0.11)	0.246	−0.06 (−0.20; 0.01)	0.078
Physical Fitness				
Hamstring flexibility	−0.02 (−0.23; 0.14)	0.635	−0.00 (−0.08; 0.07)	0.911
Speed-agility	0.06 (−0.04; 0.30)	0.135	0.06 (−0.02; 0.12)	0.151
Abdominal strength	−0.01 (−0.20; 0.17)	0.880	0.02 (−0.05; 0.09)	0.571
Lower Back strength	−0.03 (−0.25; −0.12)	0.495	0.04 (−0.04; 0.10)	0.359
Lower limb muscular strength	−0.04 (−0.30; 0.10)	0.311	0.01 (−0.06; 0.09)	0.747
VO2Max	−0.09 (−0.47; −0.04)	0.022	−0.05 (−0.15; 0.02)	0.142
Fitness Score 1	−0.04 (−0.69; 0.27)	0.394	−0.01 (−0.19; 0.17)	0.889

Values shown are β (95% CI). Abbreviations: BMI: body mass index; VO2Max: maximum oxygen volume. Linear regression models were adjusted for: age, sex, and school. Fitness score 1 was generated with the sum of the 6 physical test (z-scores), divided by 6.

**Table 3:** Odds ratio of physical fitness according to low back pain prevalence.

	Outcome Pain Frequency							
Exposures	LBP Ever (No)	*p* For Tend	LBP in the Last 7 Days (no)	*p* For Trend	LBP Today (no)	*p* for Trend	LBP in Bed (No)	*p* for Trend
Physical Activity Habits								
PAQ-A-score	0.65 (0.49; 0.87)	0.004	1.33 (0.94; 1.88)	0.105	1.42 (0.87; 2.32)	0.161	1.39 (0.91; 2.11)	0.125
Physical Fitness								
Hamstring flexibility	0.84 (0.61; 1.17)	0.300	1.03 (0.69; 1.52)	0.900	1.31 (0.75; 2.27)	0.342	1.77 (1.09; 2.86)	0.020
Speed-agility	1.23 (0.87; 1.74)	0.251	1.02 (0.67; 1.55)	0.921	0.89 (0.50; 1.59)	0.659	0.91 (0.54; 1.51)	0.706
Abdominal strength	1.00 (0.71; 1.39)	0.989	1.21 (0.81; 1.81)	0.351	1.46 (0.84; 2.54)	0.178	1.38 (0.85; 2.23)	0.189
Lower Back strength	1.11 (0.78; 1.57)	0.559	1.13 (0.75; 1.71)	0.563	1.21 (0.69; 2.14)	0.508	1.09 (0.67; 1.78)	0.736
Lower limb muscular strength	0.98 (0.70; 1.38)	0.906	1.35 (0.89; 2.06)	0.155	1.36 (0.74; 2.48)	0.322	1.30 (0.79; 2.14)	0.305
VO2Max	0.89 (0.66; 1.20)	0.434	1.25 (0.87; 1.80)	0.228	1.12 (0.67; 1.87)	0.656	1.56 (1.00; 2.42)	0.049

Values shown are odds ratios (95% CI). Logistic regression models were adjusted for age, sex, and school. Abbreviations: LBP: low back pain; BMI: body mass index. Variables categorization—Exposures: From the continuous variables, the categorical variables have been created, dividing the variables into two categories by the 50th percentile. In the present analyses, category 1, below the 50th percentile, is taken as the reference category, and the results corresponding to category 2, above the 50th percentile, are shown. The PAQ-A score was categorized <3 points (reference category) and ≥3 (shown category). Variables categorization—Outcomes: The values shown correspond to no pain, and the reference categories were the presence of pain.

## Data Availability

Not applicable.

## References

[1] Kędra A., Plandowska M., Kędra P., Czaprowski D. (2021). Non-specific low back pain: Cross-sectional study of 11,423 children and youth and the association with the perception of heaviness in carrying of schoolbags. PeerJ.

[2] Vidal-Conti J., Borràs P., Palou P., Muntaner-Mas A. Prevalence of Low Back Pain among School-Aged Children between 10 and 12 Years. https://www.mdpi.com/1346996.

[3] Miñana-Signes V., Monfort-Pañego M., Bosh-Bivià A.H., Noll M. (2021). Prevalence of low back pain among primary school students from the city of Valencia (Spain). Healthcare.

[4] Hoy D., March L., Brooks P., Blyth F., Woolf A., Bain C., Williams G., Smith E., Vos T., Barendregt J. (2014). The global burden of low back pain: Estimates from the Global Burden of Disease 2010 study. Ann. Rheum. Dis..

[5] Rezapur-Shahkolai F., Gheysvandi E., Tapak L., Dianat I., Karimi-Shahanjarini A., Heidarimoghadam R. (2020). Risk factors for low back pain among elementary school students in western Iran using penalized logistic regression. Epidemiol. Health.

[6] Klyne D.M., Hall L.M., Nicholas M.K., Hodges P.W. (2022). Risk factors for low back pain outcome: Does it matter when they are measured?. Eur. J. Pain.

[7] Minghelli B. (2020). Musculoskeletal spine pain in adolescents: Epidemiology of non-specific neck and low back pain and risk factors. J. Orthop. Sci..

[8] Dantas M.G.B., Aquino A.N., Correia H.J., Ferreira K.P., Nascimento B., Silva L.S., Da Silva A., Penha P.J., João S. (2021). Prevalence of Back Pain and Idiopathic Scoliosis in Adolescents From the Semiarid Region of Brazil: A Cross-sectional Study. J. Chiropr. Med..

[9] Kovacs F.M., Gestoso M., Gil Del Real M.T., López J., Mufraggi N., Ignacio Méndez J. (2003). Risk factors for non-specific low back pain in schoolchildren and their parents: A population based study. Pain.

[10] Auvinen J., Tammelin T., Taimela S., Zitting P., Karppinen J. (2008). Associations of physical activity and inactivity with low back pain in adolescents. Scand. J. Med. Sci. Sports.

[11] Fritz J.M., Clifford S.N. (2010). Low back pain in adolescents: A comparison of clinical outcomes in sports participants and nonparticipants. J. Athl. Train..

[12] Potthoff T., de Bruin E.D., Rosser S., Humphreys B.K., Wirth B. (2018). A systematic review on quantifiable physical risk factors for non-specific adolescent low back pain. J. Pediatr. Rehabil. Med..

[13] García-Hermoso A., Ramírez-Campillo R., Izquierdo M. (2019). Is Muscular Fitness Associated with Future Health Benefits in Children and Adolescents? A Systematic Review and Meta-Analysis of Longitudinal Studies. Sports Med..

[14] Zhu W., Mahar M.T., Welk G.J., Going S.B., Cureton K.J. (2011). Approaches for development of criterion-referenced standards in health-related youth fitness tests. Am. J. Prev. Med..

[15] Bo Andersen L., Wedderkopp N., Leboeuf-Yde C. (2006). Association between back pain and physical fitness in adolescents. Spine.

[16] Fühner T., Kliegl R., Arntz F., Kriemler S., Granacher U. (2021). An Update on Secular Trends in Physical Fitness of Children and Adolescents from 1972 to 2015: A Systematic Review. Sports Med..

[17] Bull F.C., Al-Ansari S.S., Biddle S., Borodulin K., Buman M.P., Cardon G., Carty C., Chaput J.P., Chastin S., Chou R. (2020). World Health Organization 2020 guidelines on physical activity and sedentary behaviour. Br. J. Sports Med..

[18] Borras P.A., Vidal-Conti J. (2022). An on-line school-based randomised controlled trial to prevent non-specific low back pain in children. Health Educ. J..

[19] Palou P., Kovacs F.M., Vidal J., Gili M., Borràs P.A., Gestoso M., Ponseti X. (2010). Validation of a questionnaire to determine risk factors for back pain in 10–12 year-old school children. Gazz. Med. Ital. Arch. Sci. Med..

[20] Kowalski K., Crocker P., Donen R., Honours B. The Physical Activity Questionnaire for Older Children (PAQ-C) and Adolescents (PAQ-A) Manual. https://www.researchgate.net/publication/228441462.

[21] Martínez-Gómez D., Martínez-De-Haro V., Pozo T., Welk G.J., Villagra A., Calle M.E., Marcos A., Veiga O.L. (2009). Reliability and validity of the PAQ-A questionnaire to assess physical activity in Spanish adolescents. Rev. Esp. De Salud Publica.

[22] Ruiz J., España-Romero V., Castro-Piñero J., Artero E., Ortega F., Cuenca-García M., Jiménez-Pavón D., Chillón P., Girela-Rejón M., Mora J. (2011). ALPHA-fitness test battery: Health-related field-based fitness tests assessment in children and adolescents. Nutr. Hosp..

[23] Castro-Pinero J., Chillon P., Ortega F.B., Montesinos J.L., Sjostrom M., Ruiz J.R. (2009). Criterion-related validity of sit-and-reach and modified sit-and-reach test for estimating hamstring flexibility in children and adolescents aged 6-17 years. Int. J. Sports Med..

[24] Allen B.A., Hannon J.C., Burns R.D., Williams S.M. (2014). Effect of a core conditioning intervention on tests of trunk muscular endurance in school-aged children. J. Strength Cond. Res..

[25] Biering-Sorensen F. (1984). Physical measurements as risk indicators for low-back trouble over a one-year period. Spine.

[26] Fjørtoft I., Pedersen A.V., Sigmundsson H., Vereijken B. (2011). Measuring physical fitness in children who are 5 to 12 years old with a test battery that is functional and easy to administer. Phys. Ther..

[27] Lardon A., Leboeuf-Yde C., le Scanff C. (2015). Is back pain during childhood or adolescence associated with muscle strength, muscle endurance or aerobic capacity: Three systematic literature reviews with one meta-analysis. Chiropr. Man. Ther..

[28] Constantino Coledam D.H., Aires de Arruda G., Cantieri F.P., Gomes Ribeiro E.A. (2021). Muscular fitness is associated with spinal pain among young people: A cross-sectional exploratory study. J. Bodyw. Mov. Ther..

[29] Astfalck R.G., O’Sullivan P.B., Straker L.M., Smith A.J. (2010). A detailed characterisation of pain, disability, physical and psychological features of a small group of adolescents with non-specific chronic low back pain. Man. Ther..

[30] Bernard J.C., Bard R., Pujol A., Combey A., Boussard D., Begue C., Salghetti A.M. (2008). Muscle assessment in healthy teenagers, Comparison with teenagers with low back pain. Ann. De Readapt. Et De Med. Phys. Rev. Sci. De La Soc. Fr. De Reeduc. Fonct. De Readapt. Et De Med. Phys..

[31] Jones M.A., Stratton G., Reilly T., Unnithan V.B. (2005). Biological risk indicators for recurrent non-specific low back pain in adolescents. Br. J. Sports Med..

[32] Hayden J., van Tulder M.W., Malmivaara A., Koes B.W. (2005). Exercise therapy for treatment of non-specific low back pain. Cochrane Database Syst. Rev..

[33] Perry M., Straker L., O’sullivan P., Smith A., Hands B. (2009). Fitness, motor competence, and body composition are weakly associated with adolescent back pain. J. Orthop. Sports Phys. Ther..

[34] Hides J.A., Jull G.A., Richardson C.A. (2001). Long-term effects of specific stabilizing exercises for first-episode low back pain. Spine.

[35] Behm D.G., Drinkwater E.J., Willardson J.M., Cowley P.M. (2010). Canadian Society for Exercise Physiology position stand: The use of instability to train the core in athletic and nonathletic conditioning. Appl. Physiol. Nutr. Metab..

[36] Cho H.Y., Kim E.H., Kim J., Kim E.H. (2014). Effects of the CORE Exercise Program on Pain and Active Range of Motion in Patients with Chronic Low Back Pain. J. Phys. Ther. Sci..

[37] Amit K., Manish G., Taruna K. (2013). Effect of Trunk Muscles Stabilization Exercises and General Exercises on Pain in Recurrent Non Specific Low Back Ache. Int. Res. J. Med. Sci..

[38] Mayer F., Arampatzis A., Banzer W., Beck H., Brüggemann G.P., Hasenbring M., Kellmann M., Kleinert J., Schiltenwolf M., Schmidt H. (2018). Medicine in spine exercise [mispex]—A national research network to evaluate back pain. Dtsch. Z. Fur Sportmed..

[39] Carson V., Hunter S., Kuzik N., Gray C.E., Poitras V.J., Chaput J.P., Saunders T.J., Katzmarzyk P.T., Okely A.D., Connor Gorber S. (2016). Systematic review of sedentary behaviour and health indicators in school-aged children and youth: An update. Appl. Physiol. Nutr. Metab..

[40] Padilla-Moledo C., Fernández-Santos J.D.R., Izquierdo-Gómez R., Esteban-Cornejo I., Rio-Cozar P., Carbonell-Baeza A., Castro-Piñero J. (2020). Physical Fitness and Self-Rated Health in Children and Adolescents: Cross-Sectional and Longitudinal Study. Int. J. Env. Res. Public Health.

[41] Todendi P.F., Brand C., Silveira J.F.D.C., Gaya A.R., Agostinis-Sobrinho C., Fiegenbaum M., Burns R.D., Valim A.R. (2021). de M.; Reuter, C.P. Physical fitness attenuates the genetic predisposition to obesity in children and adolescents. Scand. J. Med. Sci. Sports.

[42] Alzahrani H., Mackey M., Stamatakis E., Zadro J.R., Shirley D. (2019). The association between physical activity and low back pain: A systematic review and meta-analysis of observational studies. Sci. Rep..

[43] Gordon R., Bloxham S. (2016). A Systematic Review of the Effects of Exercise and Physical Activity on Non-Specific Chronic Low Back Pain. Healthcare.

[44] Heneweer H., Staes F., Aufdemkampe G., van Rijn M., Vanhees L. (2011). Physical activity and low back pain: A systematic review of recent literature. Eur. Spine J..

[45] Sato T., Ito T., Hirano T., Morita O., Kikuchi R., Endo N., Tanabe N. (2011). Low back pain in childhood and adolescence: Assessment of sports activities. Eur. Spine J..

[46] Kikuchi R., Hirano T., Watanabe K., Sano A., Sato T., Ito T., Endo N., Tanabe N. (2019). Gender differences in the prevalence of low back pain associated with sports activities in children and adolescents: A six-year annual survey of a birth cohort in Niigata City, Japan. BMC Musculoskelet. Disord..

[47] Guddal M.H., Stensland S.Ø., Småstuen M.C., Johnsen M.B., Zwart J.A., Storheim K. (2017). Physical Activity Level and Sport Participation in Relation to Musculoskeletal Pain in a Population-Based Study of Adolescents: The Young-HUNT Study. Orthop. J. Sports Med..

[48] Wedderkopp N., Kjaer P., Hestbaek L., Korsholm L., Leboeuf-Yde C. (2009). High-level physical activity in childhood seems to protect against low back pain in early adolescence. Spine J..

[49] Sitthipornvorakul E., Janwantanakul P., Purepong N., Pensri P., van der Beek A.J. (2011). The association between physical activity and neck and low back pain: A systematic review. Eur. Spine J..

[50] Aartun E., Hartvigsen J., Boyle E., Hestbaek E. (2016). No associations between objectively measured physical activity and spinal pain in 11-15-year-old Danes. Eur. J. Pain.

[51] Ruiz J.R., Ortega F.B., Rizzo N.S., Villa I., Hurtig-Wennlöf A., Oja L., Sjöström M. (2007). High cardiovascular fitness is associated with low metabolic risk score in children: The European Youth Heart Study. Pediatr. Res..

[52] Noll M., Kjaer P., Mendonça C.R., Wedderkopp N. (2022). Motor performance and back pain in children and adolescents: A systematic review. Eur. J. Pain.

